# Attentional Control in Adolescent Mice Assessed with a Modified Five Choice Serial Reaction Time Task

**DOI:** 10.1038/s41598-017-10112-8

**Published:** 2017-08-30

**Authors:** Mariasole Ciampoli, Gabriella Contarini, Maddalena Mereu, Francesco Papaleo

**Affiliations:** 1 Department of Neuroscience and Brain Technologies, Istituto Italiano di Tecnologia, via Morego, 30, 16163 Genova, Italy; 2 Department of Pharmacological and Pharmaceutical Science, University of Padova, Padova, Italy

## Abstract

Adolescence is a critical period for the development of higher-order cognitive functions. Unlike in humans, very limited tools are available to assess such cognitive abilities in adolescent rodents. We implemented a modified 5-Choice Serial Reaction Time Task (5CSRTT) to selectively measure attentiveness, impulsivity, broad monitoring, processing speed and distractibility in adolescent mice. 21-day old C57BL/6J mice reliably acquired this task with no sex-dependent differences in 10–12 days. A protocol previously used in adults was less effective to assess impulsiveness in adolescents, but revealed increased vulnerability in females. Next, we distinctively assessed selective, divided and broad monitoring attention modeling the human Spatial Attentional Resource Allocation Task (SARAT). Finally, we measured susceptibility to distractions using non-predictive cues that selectively disrupted attention. These paradigms were also applied to two genetically modified lines: the dopamine transporter (DAT) and catechol-O-methyltransferase (COMT) heterozygous. Adolescent DAT hypo-functioning mice showed attentional deficits and higher impulsivity as found in adults. In contrast to adults, adolescent COMT hypo-functioning mice showed decreased impulsivity and attentional resilience to distractors. These paradigms open new avenues to study the establishment of higher-order cognitive functions in mice, as well as an effective tool for drug-testing and genetic screenings focused on adolescence.

## Introduction

Adolescence is a critical transitional period of development from infancy to adulthood in which neurochemical and hormonal brain maturational processes extensively shape mammalian behaviors^1–3^. In particular, higher order cognitive functions drastically develop and mature during this time period^4^. Indeed, significant improvements in cognitive functioning are evident throughout late childhood and adolescence, with the most dramatic progress occurring in the development of attentional control, processing speed, decision making, planning, and response inhibition^5, 6^.

The development of attentional control is crucial because this ability might strongly influence all other cognitive domains^7, 8^. Attentional control abilities start to emerge in childhood^9, 10^, but their full maturation peaks during adolescence^11^. Moreover, the speed of attentional control, its accuracy, inhibitory control towards irrelevant stimuli and the ability to disengage from one focus to another greatly improve throughout adolescence^12^. Additionally, it has been observed that adolescents are more prone to risk taking behavior and impulsiveness, compared to infants and adults^13, 14^. Notably, adolescents with poorer attentional regulation have worse health, earn less money and commit more crimes during adulthood^15^. To trace the development of the above mentioned abilities from infancy, through adolescence, to adulthood, the serial reaction time task and other similar tasks have been extensively used in human studies^16–18^.

Animal models are a useful tool to identify molecular and circuital processes potentially underlying the neurobiological basis of the maturational changes observed in human adolescence. The most drastic changes in terms of neuronal architecture and function have been identified within the prefrontal cortical areas (PFC)^19–21^. For example, in the PFC, adolescent rodents show prolonged neuronal pruning^22^, a drastic maturation of the glutamatergic, dopaminergic and GABAergic systems^22–24^ and a shift in the balance between mesocortical and mesolimbic systems^25^. Similarly, human neuroimaging studies suggest that adolescence is characterized by changes in patterns of brain activation, including increased activation in ventral PFC regions^26–28^ and exaggerated accumbens activity related to rewarding outcomes compared to children or adults^29, 30^. However, despite several elegant studies dissecting the changes in brain circuits and molecular footprints in animal models, very limited behavioral tools that reliably assess higher order cognitive functions are available for adolescent rodents. Thus, there is still a significant gap between the extensive and complex human literature on cognitive development and the scarce equivalent tools in rodents. Behavioral paradigms able to selectively dissect different forms of attentional control during rodent adolescence could help clarify the dynamic changes creditably observed at the molecular level, drawing better parallelisms with human studies. Finally, because adolescence is considered to be a period of higher vulnerability and increased risk of onset for several psychiatric disorders^31^, appropriate cognitive tasks for rodents could help to discern the impact of genetic and environmental factors.

**Figure 1:**
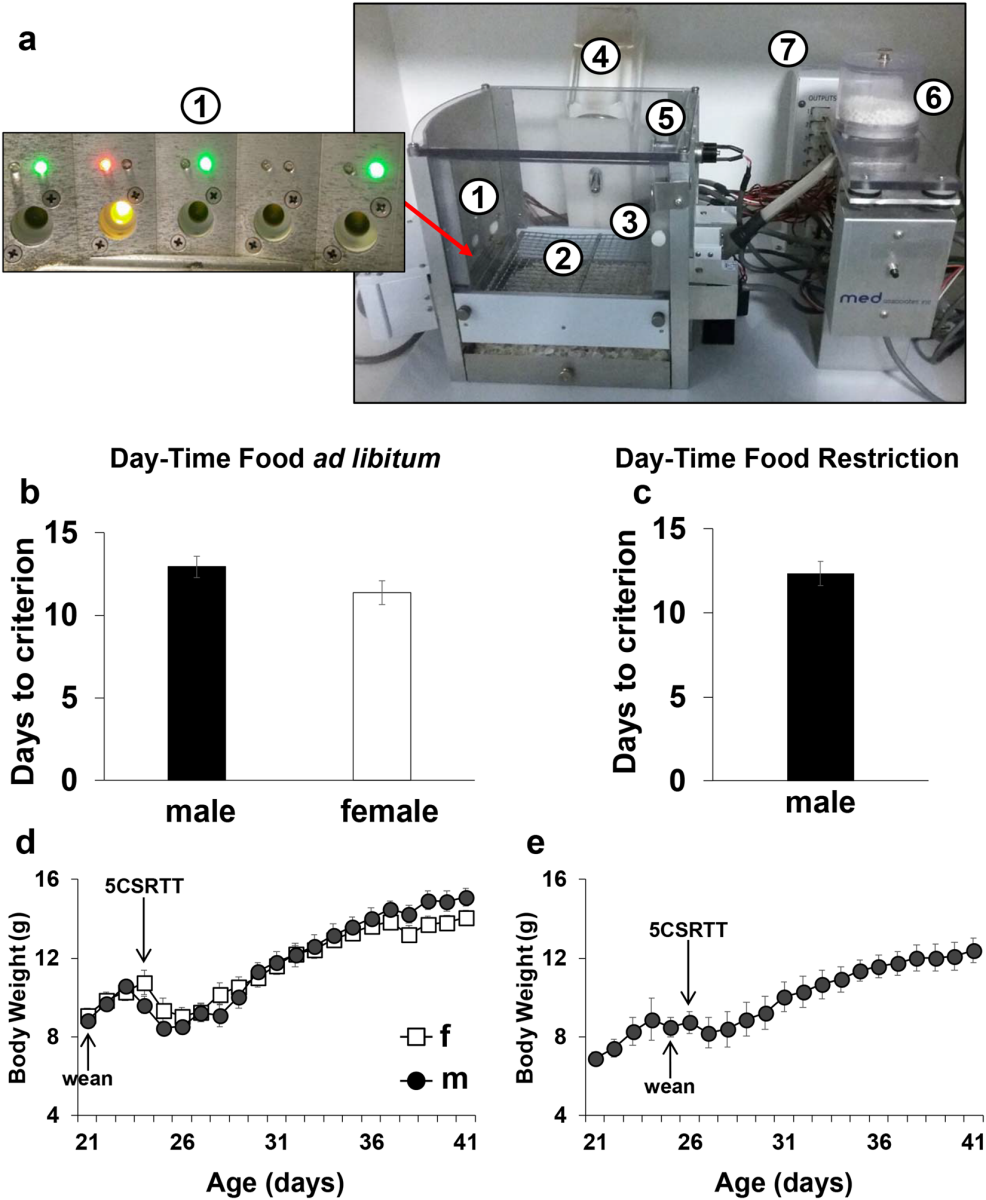
(**a**) The modified 5CSRTT apparatus: (1) modified 5 nose-poke holes wall, each outfitted with a recessed LED stimulus light and 2 additional LED cue lights (red and green) above each of the 5 nose-poke holes. (2) A stainless steel grid floor modified for the use in adolescent mice. (3) Food magazine on the wall opposite to the 5-hole array. (4) Water dispenser. (5) House-light. (6) Food pellet dispenser. (7) Smart Control Panel. (All the standard components were obtained from Med Associates, St. Albans, VT, USA). (**b**) Number of days taken by C57BL/6J male and female adolescent mice kept under food *ad libitum* condition during the light phase of the day to reach Stage 6 criteria. (**c**) Number of days taken by C57BL/6J male adolescent mice kept under food restriction condition during the light phase of the day to reach Stage 6 criteria. (**d**) Morning body weight measurements (in grams) of C57BL/6J male and female adolescent mice kept under food *ad libitum* condition during the light phase of the day. Ns = 15 males and 14 females. (**e**) Morning body weight measurements (in grams) of C57BL/6J male adolescent mice kept under food restriction condition during the light phase of the day. Ns = 6 males. Values represent mean ± SEM in all Figures.

Here we validated a modified version of the 5-Choice Serial Reaction Time Task (5CSRTT) for adolescent mice (Fig. 1a). Available tasks to assess higher-order cognitive functions have been designed and tested only in adult mice and rats^32–35^. This is mostly due to the long periods required for training, which are incompatible with the very short duration of rodent adolescence. Similarly to another recently modified 5CSRTT^36^, our task is acquired by adolescent mice in about 12 days only, in the context of no food restriction regimens. Additionally, our task is performed minimizing single-housing, since adolescence is considered to be a delicate period for the development of social skills. Moreover, this new task did not require any additional cage other than the 5CSRTT apparatus. The novel automatic paradigms implemented are effective in differentially measuring multiple attentional functions such as selective and divided attention, broad monitoring, vulnerability to distractors, impulsivity, speed of processing and motivation in adolescent mice. This was validated in both males and females as well as in two different genetically modified mouse lines (i.e. DAT and COMT), highlighting substantial divergences in performance between adolescents and adults. Combined with the advanced techniques currently available to study the impact of molecular-, circuital-, cell- and genetic-specific factors in mice, this new behavioral tool will help improve our understanding of adolescence.

## Results

### Adolescent mice readily acquired the modified 5CSRTT

In order to keep the duration of the task within the very short period of mice “adolescence” (i.e. ≈25–50 days old), the first challenge we had to face was to shorten the long training which is usually required for the classical 5CSRTT for adults^37^. The implementation of three testing sessions randomly presented during the night phase successfully decreased the time needed to acquire the task. Indeed, about 85% of mice were able to acquire the task in an average of about 12 days (Fig. 1b,c). There was no sex-dependent effect on the number of days needed to reach the final stage (F_1,27_ = 2.1, p = 0.15; Fig. 1b). Notably, at the end of the training phase, mice were still in the middle portion of “adolescence” (≈32 days old). This was achieved maintaining a normal adolescent body weight-growing curve (Fig. 1d,e). If *ad libitum* access to food was kept in the home cage during the light phase of the day, mice typically lost weight the mornings that followed the first 3 nights of testing (F_21,546_ = 3.5, p < 0.0005), but all mice quickly recovered gradually growing throughout the test (p < 0.0001; Fig. 1d). In contrast, restricting the access to food during the light phase of the day, as is usually done in adults^34, 37^, abolished the initial morning body weight loss (F_21,546_ = 134.070, p < 0.0005; Fig. 1e). Nonetheless, under the two food regime conditions, mice performance did not vary in all parameters described below, a part from the omissions (66.8 ± 2.0 or 59.7 ± 2.0 for day-time food *ad libitum* or restriction, respectively; p = 0.01). This demonstrates that the three test sessions per night were sufficient to keep the normal growing curve of adolescent mice, while ensuring a quick acquisition of the task and maintaining a good level of performance.

### The 5–7 second inter-trial interval (ITI) shift is not effective in triggering premature responses in male C57BL/6J adolescent mice

**Figure 2:**
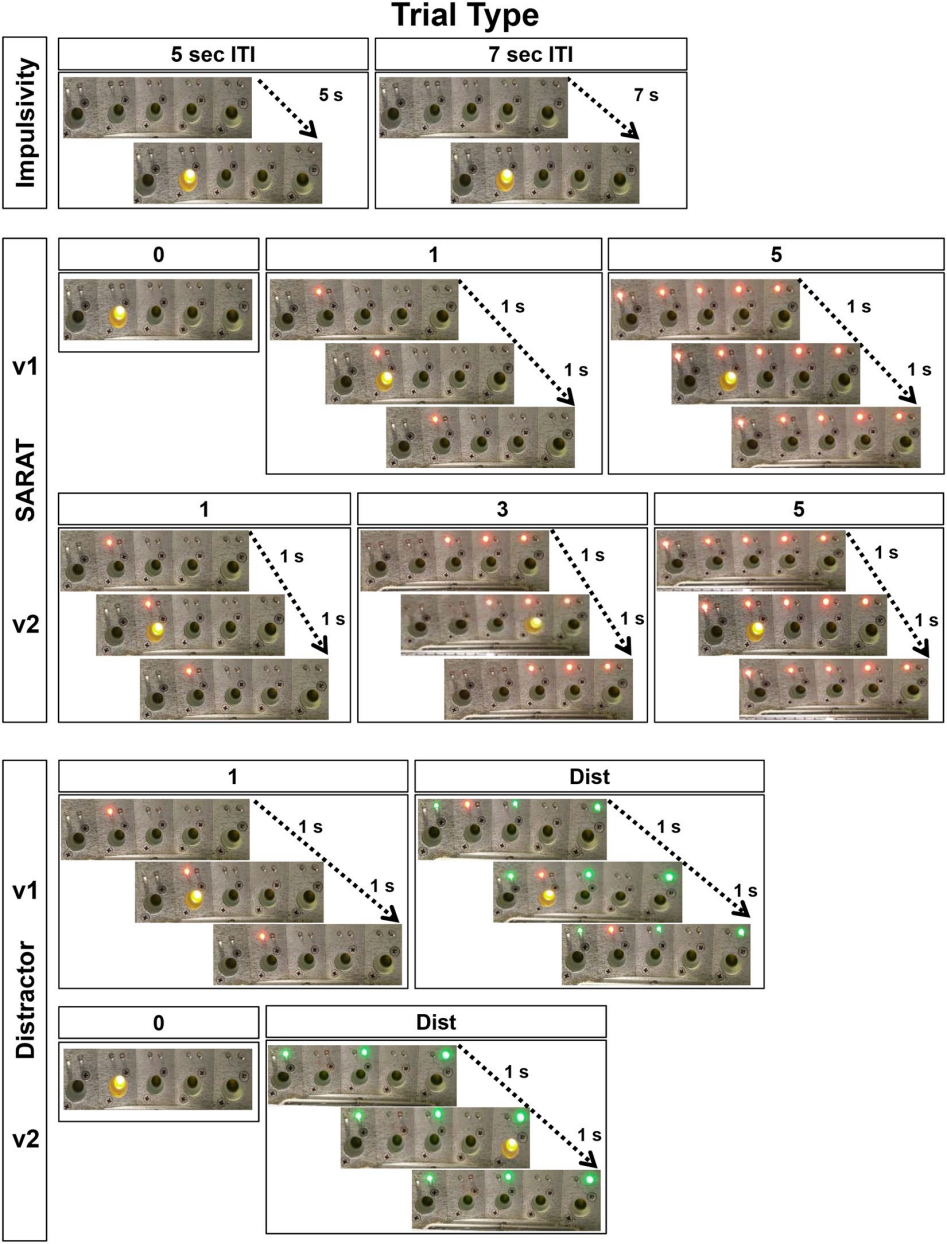
Schematic diagrams of the trials type that were presented to the mice during the three different test manipulation paradigms: Impulsivity; SARAT v1 and v2 and Distractor v1 and v2.

Upon reaching the training criteria with the basic stage of the 5CSRTT, adolescent mice were exposed to different paradigms with different trial manipulations as summarized in Fig. 2.

**Figure 3:**
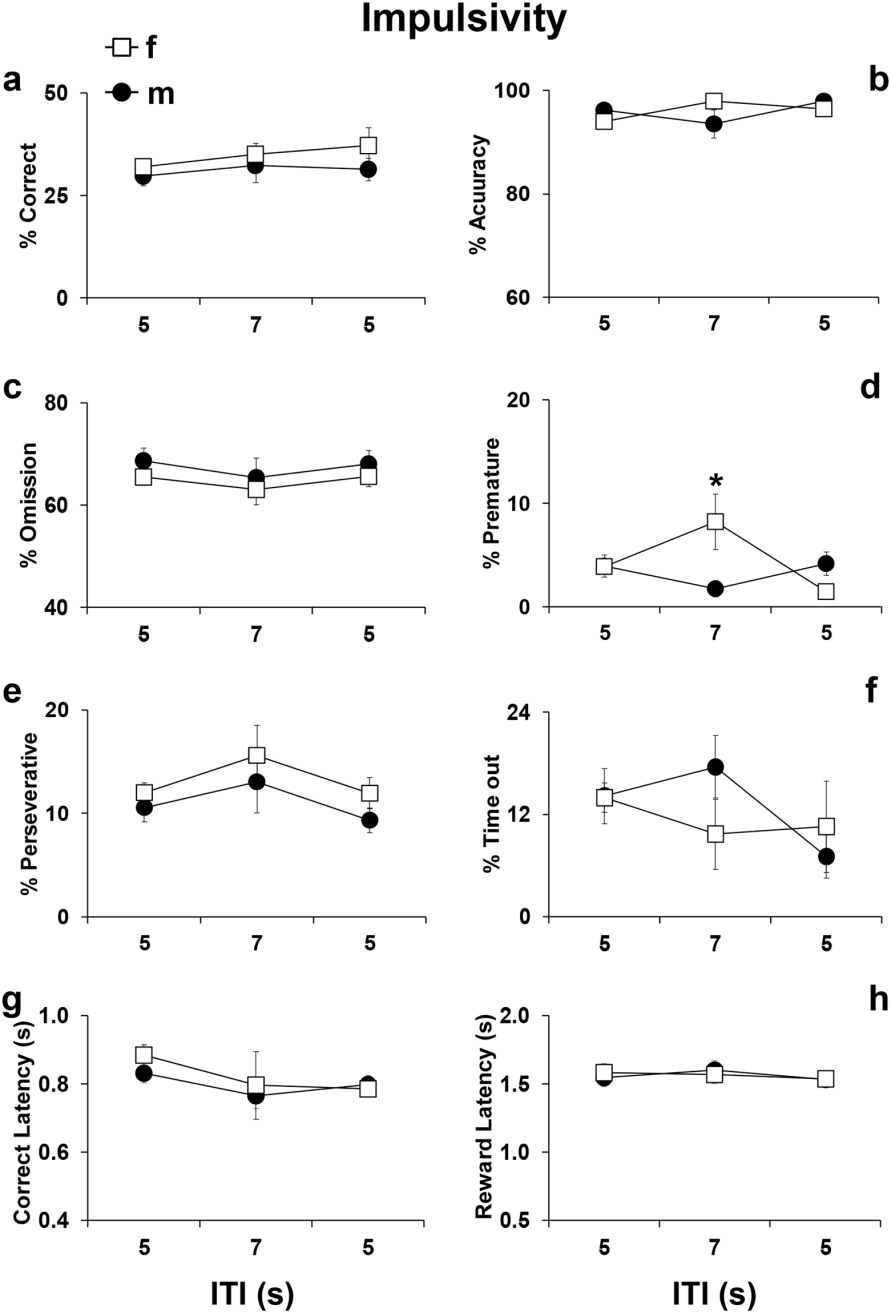
Performance displayed by C57Bl/6J male and female adolescent mice during the Impulsivity screening at different Inter-trial Interval delays (i.e. ITI of 5 or 7 seconds). Percentage of (**a**) correct responses (correct responses/total number of trials*100), (**b**) accuracy (correct responses/(correct + incorrect responses)*100), (**c**) omissions (omitted trials/total number of trials*100), (**d**) premature responses (premature responses/(correct + incorrect + premature + perseverative + time-out responses)*100), (**e**) perseverative responses (perseverative responses/(correct + incorrect + premature + perseverative + time-out responses)*100), (**f**) time-out responses (time-out responses/(correct + incorrect + premature + perseverative + time-out responses)*100), (**g**) correct latency (time in seconds from onset of light stimulus to the performance of a correct response/number of correct responses) and (**h**) reward latency (time in seconds from the performance of a correct response to the retrieval of the food reward from the food magazine/number of correct responses). Data from consecutive sessions were averaged within each trial type. For clarity, the first depicted trial type represents the performance during the previous days of only *Cued 0* trials, while the other two depicted trial types were the performance during the day of impulsivity screening. Ns = 15 males and 14 females. *p < 0.05 versus trials with a 5-second ITI. *p < 0.05 versus performance at 5-ITI trials and versus males performance at the 7-ITI trials.

The impulsivity trait in adult rats and mice becomes appreciable in the 5CSRTT when the ITI is increased from 5 to 7 seconds^34, 38^. To test whether a similar outcome could be obtained in adolescent mice, we tested in our modified 5CSRTT paradigm this same 5–7 ITI challenge (Fig. 2: impulsivity paradigm). A significant ITI-by-sex interaction effect was evident for accuracy (F_2,54_ = 4.94, p = 0.01) and premature responses (F_2,54_ = 6.78, p = 0.002). *Post*-*hoc* analyses revealed no significant effects for accuracy (p = 0.6; Fig. 3b), but an increase in premature responding in the 7-s trials in female adolescent mice (p = 0.008; Fig. 3d), but not in males (p = 0.5; Fig. 3d). As shown in Fig. 3, the 5- to 7-s ITI shift did not influence any other parameter including correct responses (F_2,54_ = 0.3, p = 0.7), omissions (F_2,54_ = 0.1, p = 0.8), perseverative responses (F_2,54_ = 0.2, p = 0.8), time-out responses (F_2,54_ = 1.58, p = 0.2), latencies to correct responses (F_2,54_ = 0.1, p = 0.8) and reward retrieval (F_2,54_ = 0.2, p = 0.8). These results indicate that this manipulation was less effective in inducing impulsive-like behaviors in adolescent mice than in adult mice. Moreover, similarly to what was reported for adult mice^34^, females showed more vulnerability to impulsivity challenges than males.

### Adolescent mice showed faster reaction time with a valid pre-cue, but difficulties distributing attention broadly

The Spatial Attentional Resource Allocation Task (SARAT) has been described as a visuospatial attention paradigm in humans able to selectively investigate broad monitoring abilities and discriminate dysfunctions in patients with psychiatric disorders such as schizophrenia^39^. Notably, visuospatial functioning is impaired in children and adolescents with psychiatric disorders such as schizophrenia, ADHD, autism and 22q11.2DS^40^. Thus, here we implemented a variation of the 5CSRTT modelled after the human SARAT protocol (Fig. 2: SARAT v1 paradigm).

**Figure 4:**
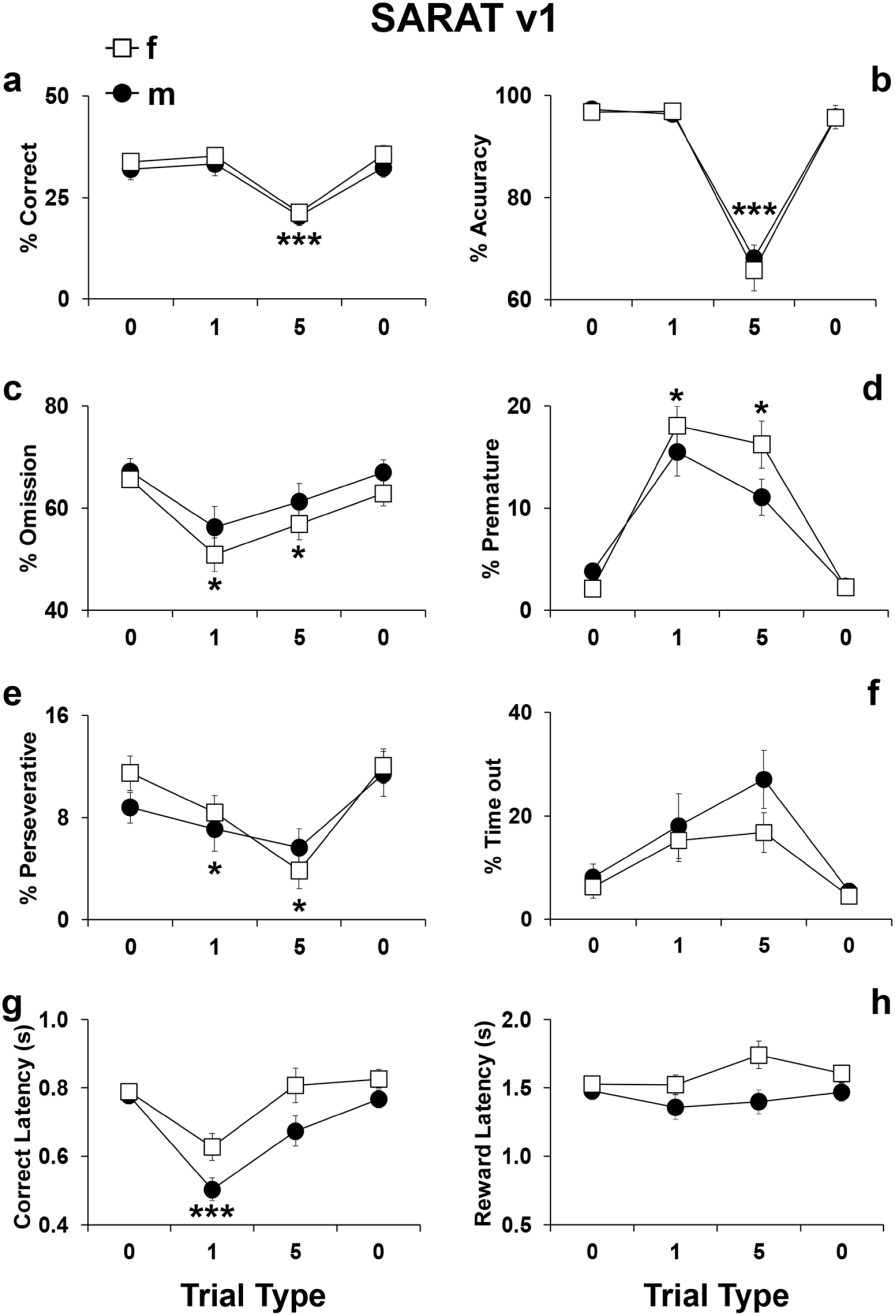
Performance displayed by C57Bl/6J male and female adolescent mice during the SARAT test version 1. Percentage of (**a**) correct responses (correct responses/total number of trials*100), (**b**) accuracy (correct responses/(correct + incorrect responses)*100), (**c**) omissions (omitted trials/total number of trials*100), (**d**) premature responses (premature responses/(correct + incorrect + premature + perseverative + time-out responses)*100), (**e**) perseverative responses (perseverative responses/(correct + incorrect + premature + perseverative + time-out responses)*100), (**f**) time-out responses (time-out responses/(correct + incorrect + premature + perseverative + time-out responses)*100), (**g**) correct latency (time in seconds from onset of light stimulus to the performance of a correct response/number of correct responses) and (**h**) reward latency (time in seconds from the performance of a correct response to the retrieval of the food reward from the food magazine/number of correct responses). Data from consecutive sessions were averaged within each trial type. For clarity, the first depicted trial type represents the performance during the previous days of only *Cued 0* trials, while the other two depicted trial types were the performance during the day of SARAT screening. Ns = 15 males and 14 females. *p < 0.05 and ***p < 0.0005 versus performance at all other trials type.

The number of cued locations defined the predictability of the target location. Only 1 cued location (i.e. *Cued 1* trials) provided a precise information about the target, allowing a narrower and more selective attentional focus. Conversely, the *Cued 5* trials increased spatial uncertainty and the need to monitor broadly. As reported in Fig. 4, a trial effect was evident for correct responses (F_3,81_ = 23.4, p < 0.0001), accuracy (F_3,81_ = 123.5, p < 0.0001), omissions (F_3,81_ = 13.13, p < 0.0001), premature responses (F_3,81_ = 54.7, p < 0.0001), perseverative responses (F_3,81_ = 18.31, p < 0.0001), time out responses (F_3,81_ = 13.54, p < 0.0001), and correct latency (F_3,81_ = 23.54, p < 0.0001). In particular, the *Cued 5* trials produced a consistent decrease in correct responses (p = 0.0001; Fig. 4a) and accuracy (p = 0.0001; Fig. 4b). Both *Cued 1* and *Cued 5﻿* trials decreased omissions (p < 0.05; Fig. 4c), increased time out responses (p < 0.005; Fig. 4f), increased premature responses (p < 0.05; Fig. 4d) and decreased perseverative responses (p < 0.05; Fig. 4e). The *Cued 1* trials selectively triggered faster correct responses (p = 0.0001 Fig. 4g). No trial effect was evident for reward latencies (F_3,81_ = 2.53, p = 0.06; Fig. 4h). Moreover, no sex-dependent effects were evident for any parameter (p > 0.4). These findings provide evidence that this SARAT paradigm can be applied to adolescent C57BL/6J mice. Indeed, as well as that of adolescent mice, the performance of healthy humans displays faster reaction times in trials with more precise pre-cues while attentional control is disrupted in trials where the pre-cues provide invalid information about the target^39, 41, 42^.

### Distracting cues selectively disrupted attentional accuracy in adolescent mice

Adolescents show less control and more distractibility during cognitive tasks that require high demand of attention^43^. Moreover, increased distractibility during adolescence has been identified as a possible risk factor for psychiatric diseases^44–46^. Thus, we developed a protocol able to assess the impact of distracting cues on the cognitive performance of adolescent mice (Fig. 2: Distractor v1 paradigm).

**Figure 5:**
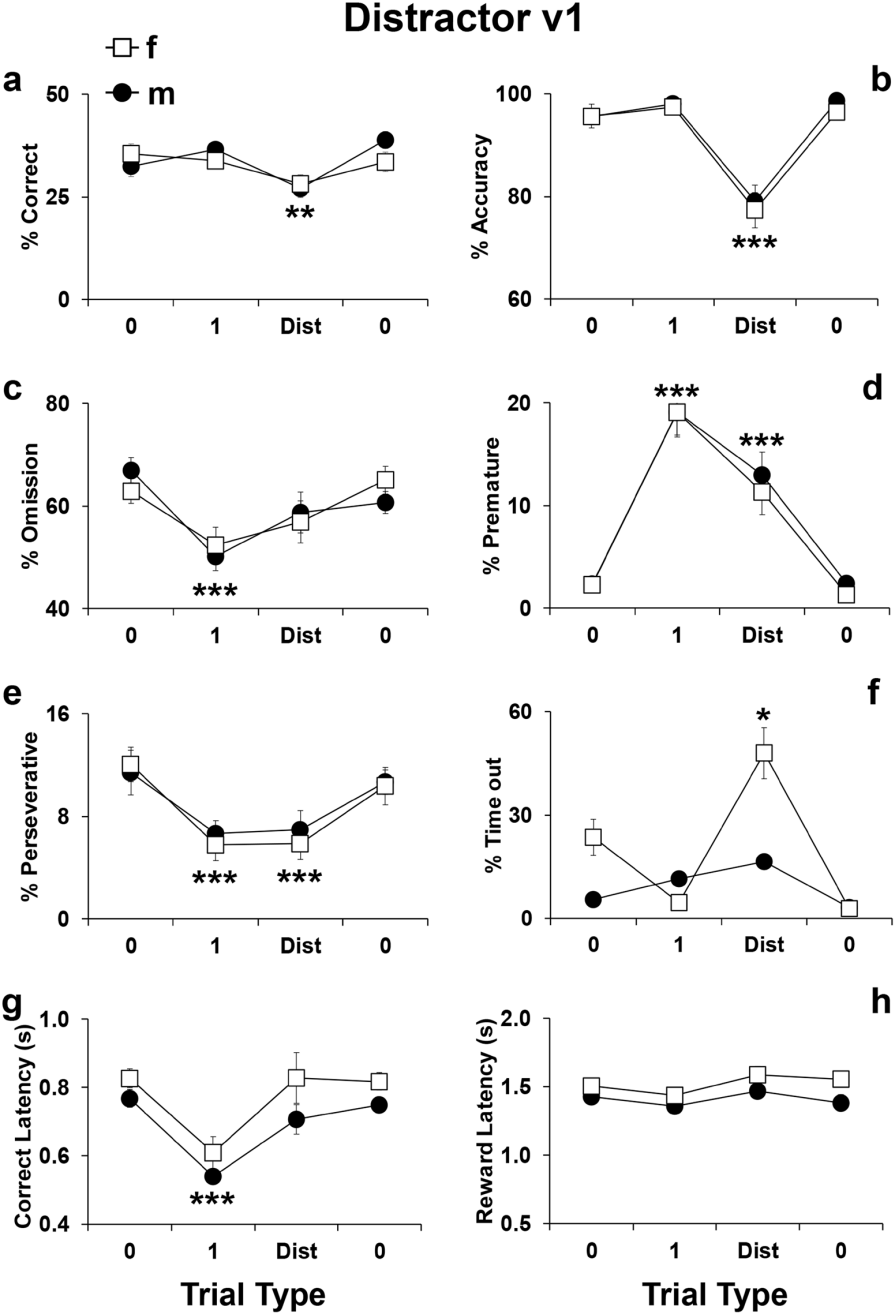
Performance displayed by C57Bl/6J male and female adolescent mice during the Distractor test version 1. Percentage of (**a**) correct responses (correct responses/total number of trials*100), (**b**) accuracy (correct responses/(correct + incorrect responses)*100), (**c**) omissions (omitted trials/total number of trials*100), (**d**) premature responses (premature responses/(correct + incorrect + premature + perseverative + time-out responses)*100), (**e**) perseverative responses (perseverative responses/(correct + incorrect + premature + perseverative + time-out responses)*100), (**f**) time-out responses (time-out responses/(correct + incorrect + premature + perseverative + time-out responses)*100), (**g**) correct latency (time in seconds from onset of light stimulus to the performance of a correct response/number of correct responses) and (**h**) reward latency (time in seconds from the performance of a correct response to the retrieval of the food reward from the food magazine/number of correct responses). Data from consecutive sessions were averaged within each trial type. For clarity, the first depicted trial type represents the performance during the previous days of only *Cued 0* trials, while the other two depicted trial types were the performance during the day of Distractor screening. Ns = 15 males and 14 females. *p < 0.05, **p < 0.05, and ***p < 0.0005 versus performance at all other trials type.

As shown in Fig. 5, a trial effect was evident for correct responses (F_3,81_ = 9.32, p < 0.0001), accuracy (F_3,81_ = 52.59, p < 0.0001), omissions (F_3,81_ = 14.68, p < 0.0001), premature responses (F_3,81_ = 61.39, p < 0.0001), perseverative responses (F_3,81_ = 14.63, p < 0.0001) and correct latencies (F_3,81_ = 23.54, p < 0.0001). In particular, trials with the distracting cues produced a decrease in correct responses (p < 0.0002; Fig. 5a) and accuracy (p < 0.0001; Fig. 5b). Both the *Cued 1* and distractor trials triggered more premature (p < 0.0001; Fig. 5d) and less perseverative responses (p < 0.0001; Fig. 5e). Finally, consistent with the SARAT results, in the *Cued 1* trials less omissions (p < 0.0001; Fig. 5c) and faster correct responses were made (p = 0.0005; Fig. 5g). A marked trial-by-sex interaction effect was evident in the time-out responses (F_3,81_ = 14.79, p < 0.0001) and reward latency (F_3,78_ = 4.84, p < 0.05). Adolescent female mice made more time-out responses (p < 0.05; Fig. 5f) and needed more time to retrieve the food pellet in the distractor trials (p < 0.05; Fig. 5h). These findings highlight the ability of the distracting manipulation to disrupt attentional control in both male and female adolescent mice.

### The SARAT and Distractor are distinct paradigms assessing selective attentional control processes

To test whether the SARAT and the Distractor paradigms could grasp distinct aspects of attentional control in adolescent mice, we further implemented these two paradigms (Fig. 2: SARAT v2 and Distractor v2 paradigms), as done in adult mice^47^. In particular, with the SARAT version 2, we directly linked mice performance to the number of presented pre-cues (i.e. 0, 1, 3 or 5) as is reported in human studies^39, 41^. Instead, in the second version of the Distractor, we eliminated the overlapping presence of the distracting green lights with the valid red pre-cue light that could generate conflicting information to the mice (see Fig. 2 for trials illustrations and comparisons).

**Figure 6:**
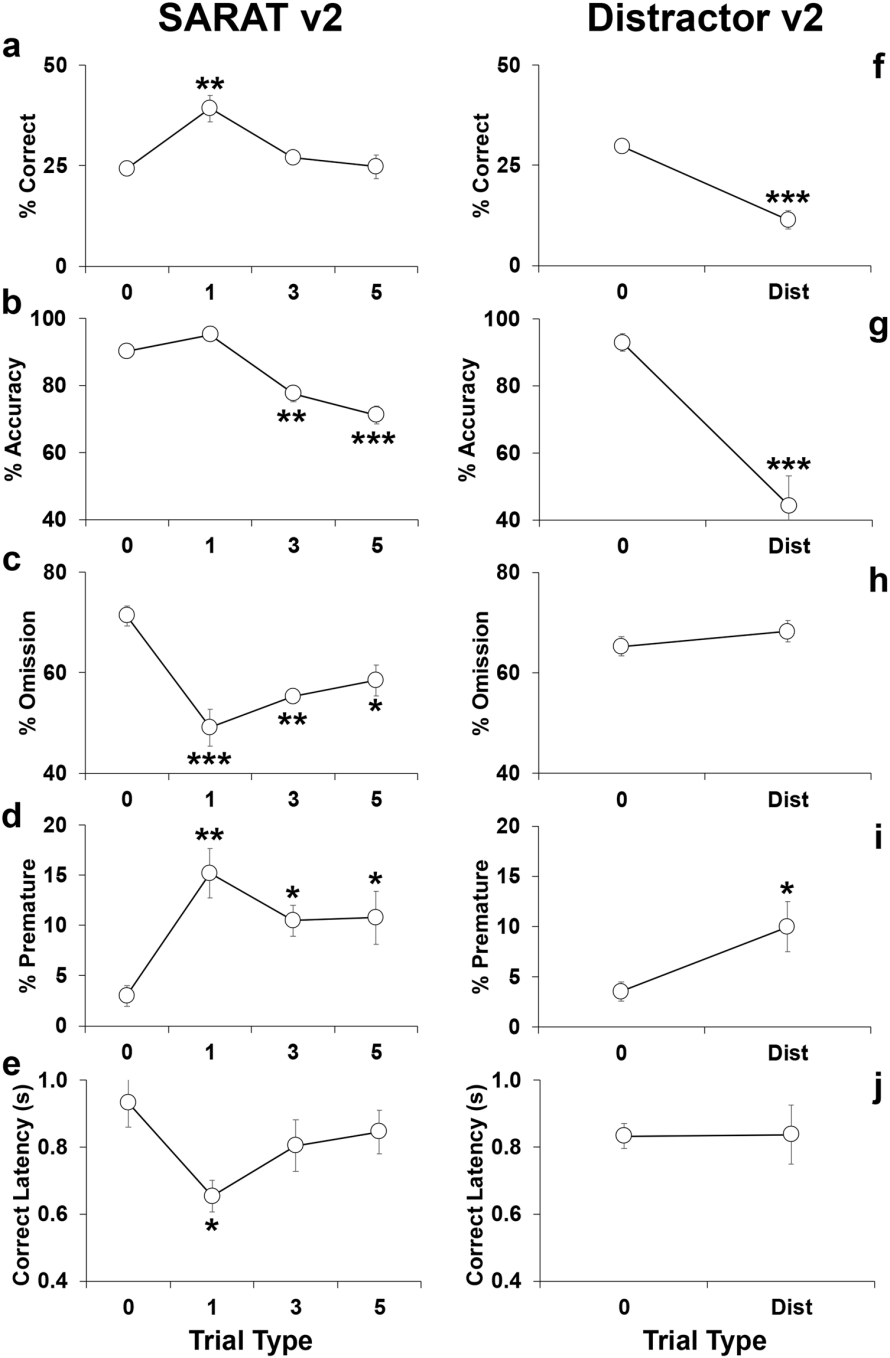
Comparison of the performance in key parameters displayed by C57Bl/6J male and female adolescent mice between the SARAT version 2 and the Distractor version 2 paradigms. Percentage of (**a** and **f**) correct responses (correct responses/total number of trials*100), (**b** and **g**) accuracy (correct responses/(correct + incorrect responses)*100), (**c** and **h**) omissions (omitted trials/total number of trials*100), (**d** and **i**) premature responses (premature responses/(correct + incorrect + premature + perseverative + time-out responses)*100), (**e** and **j**) correct latency (time in seconds from onset of light stimulus to the performance of a correct response/number of correct responses). Data from consecutive sessions were averaged within each trial type. Ns = 6 mice. *p < 0.05, **p < 0.005 and ***p < 0.0005 versus performance at all other trials type.

The SARAT v2 demonstrated that cognitive performance is tightly related to the number of valid pre-cues. Indeed, increasing spatial uncertainty to three and five pre-cues proportionately decreased the accuracy (F_3,15_ = 30.32, p < 0.0001; Fig. 6b), and increased omissions (F_3,15_ = 16.70, p < 0.0001; Fig. 6c). Instead, providing a more precise predicting cue (*Cued 1* trials) greatly ameliorated the performance of adolescent mice increasing the amount of correct responses (F_3,15_ = 9.82, p < 0.0008; Fig. 6a), decreasing the omissions (F_3,15_ = 16.70, p < 0.0001; Fig. 6c) and fastening the speed of a given correct answer (F_3,15_ = 3.28, p < 0.05; Fig. 6e). Other parameters were not altered by this manipulation.

In contrast, the distracting stimuli in the Distractor v2 decreased correct responses (t = 7.82; df = 5; p < 0.0006; Fig. 6f) and accuracy (t = −6.11; df = 5; p < 0.001; Fig. 6g) with a stronger effect compared to Distractor v1 (Fig. 5). Other parameters were not altered by this manipulation. Notably, a direct comparison between the SARAT v2 and Distractor v2 (Fig. 6 first column compared to second) highlighted the distinct pattern of performance triggered by the different stimuli. Overall, these data demonstrate that attentional control in adolescent mice can be selectively and differentially assessed by the SARAT and Distractor paradigms.

### Attentional control performance in adolescent DAT and COMT genetically hypo-functioning mice

To accentuate the effectiveness of this novel task for adolescent genetically modified mice, we tested two mice lines which we previously assessed in the 5CSRTT at adult age^47^. Specifically, we tested dopamine transporter (DAT) and catechol-O-methyltransferase (COMT) heterozygous (+/−) knockout mutant male mice, because they are clinically relevant mouse models with effects on cognitive functions that recapitulate the effects of similar genetic variations in humans^35, 48, 49^.

**Figure 7:**
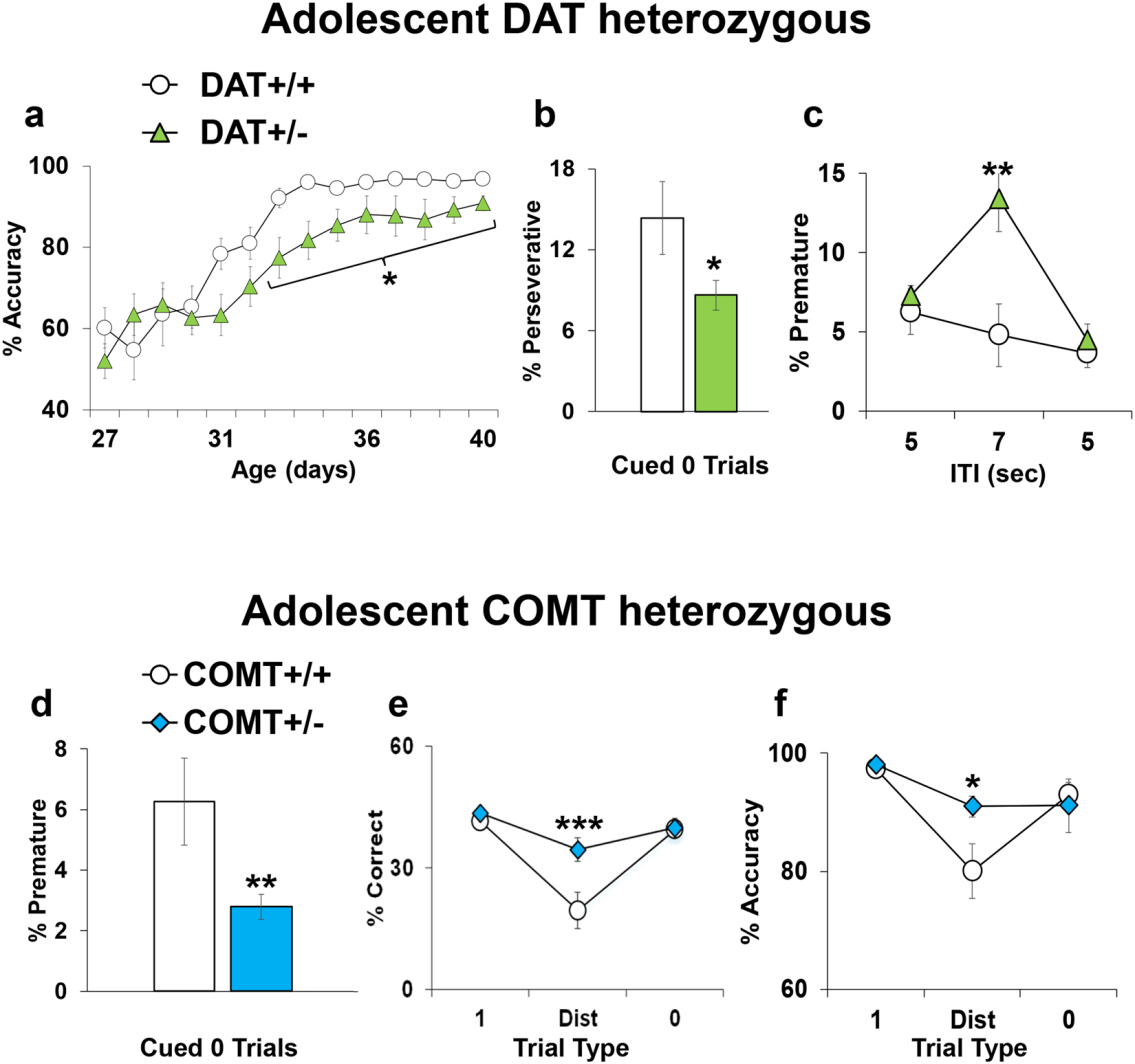
(**a**–**c**) Performance displayed by DAT+/+ and DAT+/− adolescent littermates in the modified 5CSRTT in key parameters which showed a genotype effect. (**a**) Percentage of accuracy (correct responses/(correct + incorrect responses)*100) during the training phase of the test. (**b**) Percentage of perseverative responses (perseverative responses/(correct + incorrect + premature + perseverative + time-out responses)*100) during the basic phase of the test with only trial type 0 without any extra cue. (**c**) Percentage of premature responses (premature responses/(correct + incorrect + premature + perseverative + time-out responses)*100) during the 5–7 ITI challenge paradigm. DAT+/+ Ns = 12, DAT+/− Ns = 12. *p < 0.05 and **p < 0.005 versus performance of DAT+/+ at the same trial type. (**d**–**f**) Performance displayed by COMT+/+ and COMT+/− adolescent littermates in the modified 5CSRTT in key parameters which showed a genotype effect. (**d**) Percentage of premature responses (premature responses/(correct + incorrect + premature + perseverative + time-out responses)*100) during the basic phase of the test with only trial type 0 without any extra cue. (**e**) percentage of correct responses (correct responses/total number of trials*100) and (**f**) accuracy (correct responses/(correct + incorrect responses)*100) during the Distractor paradigm. Ns: COMT+/+ = 7 and COMT+/− = 8. *p < 0.05, **p < 0.005 and ***p < 0.0005 versus performance of COMT+/+ at the same trial type.

The performance of DAT+/+ and COMT+/+ wild-type littermates followed an identical pattern of performance as that of the C57BL/6J mice shown in Figs 1–5. In contrast, compared to +/+ littermates, DAT+/− adolescent mice showed reduced accuracy during the training phase of the task (F_2,29_ = 4.37, p < 0.02; Fig. 7a), reduced levels of perseverative responses in the basic cued 0 trials (t = 2,29; df = 17; p < 0.04; Fig. 7b), and increased premature responding following the 5–7 ITI challenge (F_4,48_ = 3.33, p < 0.01; Fig. 7c). No other DAT-dependent effects were evident in any of the other parameters in all other paradigm manipulations (data not shown). Consistent with data from adult mice^48^, these results highlight that genetic variations reducing DAT produced attentional and impulsive control deficits since adolescence. Notably, despite the 5–7 ITI shift was confirmed to be ineffective in wild-type mice, it triggered a consistent increase in premature responding in DAT+/− mice, suggesting that this challenge is still effective in vulnerable subjects. Finally, an unexpected DAT effect in reducing compulsive-related phenotypes during adolescence was detected.

Instead, compared to their +/+ littermates, COMT+/− adolescent mice showed reduced levels of premature responses in the basic *Cued 0* trials (t = 2,31; df = 14; p < 0.05; Fig. 7d), and increased correct responses (F_2,26_ = 8.54, p < 0.005; Fig. 7e) and accuracy (F_2,26_ = 3.64, p < 0.05; Fig. 7f) in the distractor trials. No other COMT-dependent effects were evident in all other parameters in the other paradigm manipulations (data not shown). In contrast to what was found in adult mice^34^, these results highlight that genetic variations reducing COMT are associated with reduced levels of basal impulsivity, and an attentional control that is more resilient to the detrimental effects of distracting cues in adolescence.

## Discussion

The data reported here demonstrate that this modified 5CSRTT can effectively test attentional control abilities in adolescent mice. Moreover, different challenges in the test were able to detect in adolescent mice: (i) impulsive-like behaviors defined as the ability to refrain to make a preponderant response, (ii) the ability to maintain focused or broad attention when different pre-cue stimuli were presented (SARAT) and (iii) the attentional vulnerability to distractors.

While developing the task, preserving the chance to train mice in less than 12 days was crucial for the effectiveness of the task itself. Training in the 5CSRTT for adult mice usually requires from thirty days up to several months^34, 50, 51^. However, paradigms longer than twenty days would exceed the rodents’ short “adolescence” period which is considered to span from about 28 to 45 days of age^1, 52^. Notably, we were able to achieve this also maintaining the physiological curve of adolescent-growing body size, limiting the amount of stress and potential metabolic deficits that could derive from scarce food intake during this developmental period. Indeed, most of similar operant-based tasks in adult rodents require a food restriction protocol^34, 37, 53^. However, adolescence is a peculiar period for the vulnerability related to nutritional factors^23^. For example, an increasing body of literature illustrates a direct connection between an appropriate nutrition during adolescence and optimal cognitive and brain function^54–56^. Therefore, this novel paradigm can assess higher order cognitive functions such as attention, compulsivity, impulsivity, distractibility, decision making and processing speed in adolescent rodents with very few confounding factors. There is very scarce evidence regarding complex cognitive tasks designed for adolescent rodents. For example, intra-/extra-dimensional (ID/ED) set-shifting tasks or a two-choice visual discrimination task (2-CVDT) have been used in adolescent rats^57, 58^. However, in the ID/ED task, adolescent rats were also impaired in basic compound discrimination and in such studies food restriction was applied with no regard to the normal body weight growth of this developmental period. More recently, an all-day and self-pace testing in a similar 5CSRTT have been tested for adolescent mice^36^. In contrast to our setting, the latter task did not reveal any difference in performance of adolescents compared to adults, needed an additional cage attached to the 5CSRTT apparatus, required continuous single-housing, and its testing schedule differed from the one used in humans which is restricted in a consecutive and limited time period. This latter factor is critical when assessing sustained attention as the self-pace regime greatly reduce the attentional load. To note, we are not aware of other similar studies using distracting cues in adolescent mice. However, we chose extra visual cues (i.e. green flashing lights) randomly presented within the same session to more directly compare attentional performance with that of non-distractor trials, in order to avoid potential habituation processes found with noises^37^ and confusion with the use of house lights^59^ as reported in adults. Finally, as also previously discussed^47^, we were able to demonstrate clear differences between the SARAT and distractor paradigms. Briefly, the combination of tasks used in the current work show that the cue lights were not simply treated as target stimulus lights, as only non-predictive cues decreased the accuracy and that this was directly proportional to the degree of unpredictability (e.g. 3 vs 5 vs distracting cues). The fact that accuracy and the speed of making a correct response were both directly and proportionately modulated depending on the number of valid pre-cues presented also suggested that the mice used the cues to orient attention in anticipation of a target, and that there was a difference in this process between predictive and non-predictive cues. Moreover, our data demonstrate that faster reaction time for correct responses in cued trials were not a reflection of trials with responses initiated by the cue and executed after the target light came on. Indeed, the speed of correct responses was proportional on the number of cues presented (*Cued 1* > *Cued 3* > *Cued 0* and *5* trials), and distracting cues did not trigger faster reaction responses compared to *Cued 0* trials. Overall, all the characteristics of our modified automatic task makes it well suitable to dissect different attentional control processes in adolescent rodents also for large genetic or pharmacological screenings. This could be relevant in the context of testing early intervention/pharmacological strategies while also understanding their mechanisms. Indeed, early intervention on cognitive deficits could potentially be more effective in mitigating or reversing pathological trajectories and ameliorate the quality of life of individuals at risk for psychiatric disorders^60^.

With three different variations, we were able to selectively measure in adolescent mice subtypes of attentional control such as impulsivity, focused or broad attentiveness, processing speed and distractibility. In the “impulsivity” paradigm, adolescent female mice, but not males, increased the premature responses impulsivity index when the ITIs were changed from 5 to 7 seconds. Previous literature using delay-discounting tests found that both male and female adolescent rats exhibited greater levels of impulsive-like behaviors compared to adults^61^. However, other evidence accounted for a substantial impact of hormones in producing sex-dependent differences in impulsive actions in rodents^62^. Moreover, it has been demonstrated that in delay-discounting tasks under mild food restriction, adult female mice are more impulsive than males^63^. Premature responses are thought to reflect a failure of inhibitory response control that occurs when preparatory response mechanisms are disrupted^64, 65^. Thus, the 5-to-7 ITI shift in adolescent mice might be applied to assess sex-dependent vulnerability to this kind of impulsive control. Notably, our protocol offers another option to study impulsive control in mice. In particular, in contrast with the 5–7 ITI shift, the pre-cued trials of the SARAT protocol triggered a consistent increase in premature responding in both male and female mice. This kind of motor impulsivity is qualitatively different from the one triggered by the increase in ITI. Indeed, the pre-cue visual stimuli put forth a pre-potent response, which the mice must withhold from making in order to receive a food reward and then make a correct response. Thus, this measure of motoric impulsivity is potentially analogous to “false alarm” errors made in corresponding human tasks. Both these manipulations might constitute a valuable tool to assess impulsive behaviors in adolescent mice.

In the SARAT paradigm, adolescent mice showed a decreased accuracy in the trials where all the red cue lights were turned on (*Cued 5*), while faster speed of processing for target cues were evident in trials with more precise pre-cues (*Cued 1*). This pattern of performance was very similar to that of human healthy subjects tested in the original SARAT, where faster reaction times are evident in trials with more precise pre-cues, while cognitive performance is disrupted in trials where the pre-cues provide invalid information about the target^39, 41^. Thus, this SARAT paradigm might be useful to distinguish deficits in selective attention from deficits in broad monitoring in adolescent mice with good translational validity concerning human studies. In particular, this could be relevant for schizophrenia, as patients demonstrate more selective attentional deficits when broad focus of attention is required, rather than when attention must be focused narrowly^41^. To date, no study specifically assessed such kind of abilities in adolescent mice, making this an additional tool in preclinical investigations designed to specifically manipulate spatial selective attention.

In the Distractor test, we observed a selective disruption of attentional accuracy and increased time out responses in the distractor trials, where non-predictive flashing lights were turned on. Adolescence is considered to be a time during which many aspects of behavior including planning, multitasking and the ability to resist distractions, are profoundly shaped^66^. For instance, teenagers have more difficulties to concentrate and are easily distracted^43^. The maturation in the resistance to distractors has been associated with a decreased activation in the superior frontal sulcus between childhood and adulthood^67^, possibly linked with developmental changes in grey matter architecture and long-range connections^68^. In particular, it seems like cortical brain regions are not fully developed in humans up to the late twenties or even the early thirties, which is much later than previously thought^43^. Thus, in adolescent mice as well as in humans, it would be important to unravel the mechanisms of cognitive vulnerability to distractors, and our modified 5CSRTT might constitute a valid tool in this respect.

A comparison between adolescents’ and adults’ performance in an equivalent task might highlight interesting developmental peculiarities. Overall, the performance of adolescent mice here described was similar to that of adult mice tested in an identical 5CSRTT^47^, with few important exceptions. In particular, the sex-dependent differences in adults showing better performances in females compared to males^47^ were not evident in adolescent mice. This might reflect long-lasting effects of the sexual hormonal changes that start to appear during puberty^69–71^, and that are thought to play a critical role in the adult maturation of the cortex and complex cognitive behaviors^70, 72^. In line with this and again in contrast with responses in adults^34, 38^, we did not find any effect in C57BL6 male mice in premature responses when the ITIs were changed from 5 to 7 seconds. Furthermore, adolescent COMT+/− males showed decreased levels of premature responses (Fig. 7), while adult COMT+/− have been reported to have increased levels of premature responses^34^, even if a direct comparison with the same 5CSRTT version is still missing. However, these effects parallel recent findings unraveling a divergent dopaminergic maturation of the PFC from adolescence to adulthood between males and females^70^. Moreover, these findings raise the intriguing possibility that the COMT-dependent impact on stress vulnerability in terms of cognitive responses (e.g. impulsivity as in ref. 34) might develop in male subjects only after adolescence. This adds to previous evidence reporting that COMT-by-sex interacting effects are noticeable only between puberty and menopause^70, 73, 74^ and with data reporting a different maturation of the dopaminergic system in males compared to females^70, 75–77^. Notably, the SARAT paradigm with only one predictive cue produced attentional advantages in adolescent mice that were not evident in adults. Indeed, the increase in correct responses and the decreased omissions seen in adolescents (Fig. 6) were not apparent in adults^47^, indicating an higher attentiveness to extra-cues in adolescents. Other developmental differences in the 5CSRTT performance were then evident in the distractor paradigm, as distractor trials triggered larger deleterious effects in adolescents than in adults. Indeed, additional parameters (i.e. premature, perseverative and time out responses) were altered in adolescent mice other than just accuracy and correct choices as in adults^47^, and accuracy level was diminished to ≈40% in adolescents in contrast with ≈70% in adults. This might be related to human findings reporting higher vulnerability to distraction in adolescent subjects compared to adults^45^. Finally, in line with human findings^70, 78^, the better performance of COMT+/− in the distractor trials suggests that this manipulation might be a more sensible tool in order to highlight the cognitive advantages of COMT genetic reduction in males that were difficult to assess with classical 5CSRTT^34^. Future studies might want to address trial-by-trial analyses in order to address whether adolescents might emotionally respond differently from adults following correct or incorrect responses.

In conclusion, our results demonstrate that even within the brief duration of rodent adolescence, it is possible to assess different attentional control facets by a modified 5CSRTT paradigm. Indeed, the adopted manipulations allowed to assess different subtypes of attentional control including impulsivity, focused or broad attentiveness, processing speed and distractibility. These features suggest that this task could be a useful tool with potential translational validity concerning human studies, applicable to genetic and pharmacological studies in mouse models relevant to cognitive abnormalities and psychiatric disorders.

## Materials and Methods

### Mice

All procedures were approved by the Italian Ministry of Health (permit n. 230/2009-B) and local Animal Use Committee and were conducted in accordance with the Guide for the Care and Use of Laboratory Animals of the NIH and the European Community Council Directives. The time period defined as “adolescence” is individually variable, but it generally corresponds to the onset of puberty (from about 9–12 to 15–17 years old in humans; from about 28 to 45 days old in rodents^1, 52^. We used in-house bred mice within the range of 21–45 days old C57BL/6J (a total of 19 males and 16 females), or genetically modified (12 DAT+/+, 12 DAT+/−, 7 COMT+/+ and 8 COMT+/−) littermates. Every other generation new C57BL/6J breeders bought from Charles River were used for the C57BL/6J colony, while the lines of genetically modified mice were backcrossed with C57BL/6J for at least 10 generations. The COMT and DAT colonies were the same as described in refs 48 and 70. The breeding scheme used to obtain the genetically modified mice involved mating a+/− heterozygous male with C57BL/6J females, in order to avoid altered maternal behavior. Experimenters were blind to genotype during testing. Mice were weaned at 21 or 26 postnatal day (PND), separated for sex and housed 2–4 per cage. Mice were housed in a climate-controlled animal facility (22 ± 2 °C) and maintained on a 12-hour light/dark cycle (light on: 7 am–7 pm). All behavioral tests were conducted during the dark phase of the cycle.

## Apparatus

12 operant chambers (Med Associates, St. Albans, VT, USA), housed in sound-attenuating boxes each containing a fan for ventilation and constant background noise were used (schematics in Fig. 1a). Two strings of LED lights (one providing warm light and one providing cool light) were installed onto the ceiling of each of the sound-attenuating boxes controlled by a timer so that the 12-hour light/dark cycle was regulated (9 Lux when on). Each operant chamber contains, on 1 wall, 5 nose-poke holes (1 cm in diameter) that were each outfitted with a recessed stimulus light. Two additional LED pre-cue lights (red and green) were installed above each of the 5 nose-poke holes. An infrared beam transecting the aperture of each hole detected nose-pokes. Placed on the wall opposite to the 5-hole array, was a food magazine with an infrared beam and a head entry detector, where a pellet dispenser (ENV-203-14P) delivered food reinforcement in the form of a reward pellet (14 mg 5TUL Purified rodent tablet, TestDiet). Such reward pellets are designed to be a complete diet for the animals. A water dispenser into each operant chamber ensured full access to water throughout the training/test sessions. A house-light was located 7 cm above the food magazine. The operant chambers were connected to a Smart Control Panel and interfaced to a Windows computer equipped with a MED-PC IV software (Med Associates, St. Albans, VT, USA).

### Experimental design

#### Habituation

We tested different habituation protocols in order to check whether the weaning timing or food regimen could influence the task performance. In particular, the weaning was done or at 21 or 26PND. From PND 21 to PND 23 mice were daily exposed to 1-min handling session, given ten 14-mg pellets of the 5TUL diet and weighted. Training was started or at 24 or 27 PND. When in the testing cage, mice received food in the form of pellets (5TUL Purified rodent tablet, Test Diet). Water was always *ad libitum*. With the “day-time food *ad libitum*” regime, mice received their normal food *ad libitum* when in the regular holding cage. In contrast, with the “day-time food restriction” regime, mice were not given access to the food when in their holding cage unless losing weight, in which case extra food was provided during the day in order to keep the mice at their normal body weight curve of adolescent growth. Such food regimens were kept throughout the entire test.

#### Training protocol

Throughout training and testing, mice were daily placed into the operant chambers in the evening between 5 and 5:30 pm and taken out of the chambers the following morning between 10 and 10:30 am to be placed back into their regular holding cages (grouped house as weaned). Each night (between 7 pm and 7 am), mice were presented with three testing sessions semi-randomly and automatically presented (with a variable delay between sessions of 2–5 hours). Mice were weighed every day in the morning immediately after being taken out of the apparatus. A free reinforcement pellet was delivered at the start of each testing session. When a head entry was detected, the first trial began with an inter-trial interval (ITI). Any nose-poke during the ITI was recorded as premature response resulting in a time-out period with the house-light turned on. At the end of the time-out, the house-light was turned back off and the ITI restarted. Any nose-poke during the time-out reset the time-out period. At the end of the ITI, the program randomly selected a stimulus location (1 out of 5 stimulus lights) and turned on the corresponding stimulus light. The stimulus light remained on for the stimulus duration (SD) value set. The animal had limited hold time (LH) to nose-poke into the lit hole. A nose-poke into the lit hole during the LH, was recorded as a correct response, the stimulus light turned off if not turned off earlier and a food pellet was delivered in the opposite-wall food magazine. A nose-poke into any of the other apertures was recorded as an incorrect response. Errors resulted in the initiation of a 5-sec time out (TO) phase, during which the house light switched on and all holes were unresponsive. A lack of response within the LH period, was deemed as omission and resulted in a time-out and no reward. Premature responses (occurring in the ITI before presentation of the trigger light stimulus) also led to a time-out without reward and to a resetting of the trial. A perseverative response was scored when mice continued to poke in the same response hole when it no longer stood for a correct choice. Time from the onset of the light stimulus to the performance of a correct nose-poke response and from the correct response to the retrieval of the food reward from the magazine were recorded as correct latency and reward latency, respectively. Training consisted of 6 stages. To proceed to each subsequent stage, mice were required to reach the criterion for 2 consecutive sessions. Each stage was more challenging than the last, with the SD and LH period decreasing while other criteria become more demanding (see below). Sessions ended after 30 minutes or 100 trials, whichever comes first. Criteria to reach each subsequent stage:

1. Stage 1 to 2: SD = 20 s; LH = 30 s; ITI = 2 s.
  Criteria: ≥20 correct trials; ≥20% correct.
2. Stage 2 to 3: SD = 10 s; LH = 30 s; ITI = 2 s.
  Criteria: ≥30 correct trials; ≥30% correct.
3. Stage 3 to 4: SD = 8 s; LH = 20 s; ITI = 5 s.
  Criteria: ≥40 correct trials; ≥80% accuracy; ≤60% omission.
4. Stage 4 to 5: SD = 4 s; LH = 10 s; ITI = 5 s.
  Criteria: ≥40 correct trials; ≥80% accuracy; ≤60% omission.
5. Stage 5 to 6: SD = 2 s; LH = 7 s; ITI = 5 s.
  Criteria: ≥45 correct trials; ≥80% accuracy; ≤60% omission.
6. Stage 6: SD = 1 s; LH = 7 s; ITI = 5 s.

Upon reaching Stage 6, mice were subjected to an extra day of testing at Stage 6. After that, mice were tested with three different test protocols with in between a day of Stage 6 as explained below and in the timeline (Fig. 1a). The following measures were recorded to assess task performance as previously described^34, 37^.

Accuracy: number of correct responses divided by sum of number of correct and incorrect responses, multiplied by 100.

Correct responses: number of correct responses divided by total number of trials, multiplied by 100.

Omissions: number of omissions divided by total number of trials, multiplied by 100.

Premature responses: number of premature responses divided by sum of correct, incorrect, premature, perseverative and time-out responses (total number of responses), multiplied by 100.

Perseverative responses: number of perseverative responses divided by total number of responses, multiplied by 100.

Time-out responses: number of time-out responses divided by total number of responses, multiplied by 100.

Correct latency: total time from onset of light stimulus to the performance of a correct response divided by number of correct responses.

Reward latency: total time from the performance of a correct response to the retrieval of the food reward from the food magazine divided by number of correct responses.

#### 5–7 ITI challenge

During the 3 sessions of the night, randomly, in a 20% of the trials the ITI was increased from 5 to 7 seconds. This implicated that mice must withhold an additional 2 seconds both before the appearance of the stimulus light and before making their correct choice. The SD and LH remain unchanged.

#### Spatial Attentional Resource Allocation Task (SARAT)

Two versions of the SARAT test were performed: SARAT v1 and v2 (Fig. 2 for a representative scheme). In SARAT v1 in each of the 3 sessions during the night, three different trial types were randomly presented: *Cued 0*, as stage 6. *Cued 1*, as stage 6 but with the addition of a red cue light appearing over the correct nose-poke hole from 1 s before to 1 s after the normal stage 6 yellow stimulus light. *Cued 5*, as stage 6 but with the addition of a red cue light appearing over each nose poke hole from 1 s before to 1 s after the stage 6 yellow stimulus light. Also in the SARAT v2 three different types of trials were randomly presented. *Cued 1* trial as for SARAT v1. The *Cued 3* trial was the same as the standard trial type with the addition of 1 red pre-cue light appearing over the correct nose-poke hole and 2 pre-cue red lights appearing over the 2 nose-poke holes adjacent the correct nose-poke hole from 1 s prior to 1 s after the stimulus light duration. The third type of trial was the *Cued 5* trial as for SARAT v1. Each trial type was presented an equal number of times in a random fashion throughout each session.

#### Distractor test

In this manipulation, two versions of the Distractor test were performed: distractor v1 and v2 (Fig. 2 for a representative scheme). In v1 2 different trial types were randomly presented. *Cued 1* (80% of the time) like for the SARAT test. The *Distractor* (*Dist*) trial (20% of the time) was identical to the *Cued 1* with the addition of three green cue lights flashing from 1 second before to 1 second after the normal stage 6 yellow stimulus light. In Distractor v2, the Cued 0 trial (presented 80% of the time within a session) was the standard trial type as in Stage 6. The Distractor trial occurred 20% of the time and was the same as the Cued 0 trial with the addition of a flashing green pre-cue light over the nose-poke holes number 1, 3, and 5. In Distractor v2 no predictive pre-cue red lights were used. The green pre-cue light over the nose-poke holes were turned on from 1 s prior to 1 s after the stimulus light duration. Any nose-poke that occurred while the red/green pre-cue lights were lit, but before the normal stimulus light was presented, was considered a premature response and was not rewarded, resulting in a time-out.

### Statistical Analysis

Results are expressed as mean ± standard error of the mean (SEM) throughout. One- or two-way analyses of variance (ANOVAs) with sex (male or female) or genotype (+/+ or +/−) as between subjects factors and trial type as the within-subject repeated measure was used to analyze each single parameter measured (body weight, % Correct, % Accuracy, % Omission, % Time out, % Premature, % Perseverative, Correct latency and Reward latency). Newman-Keul’s *post-hoc* test with multiple comparisons corrections was used for making comparisons between groups when the overall ANOVA showed statistical significant differences for the main factors or interactions. Student’s t-test was used to compare the days needed to reach the criteria between males and females, the % Perseverative between DAT+/+ and +/−, the % Premature between COMT+/+ and +/−. The accepted value for significance was p < 0.05. All statistical analyses were performed using the Statistica version 12 software (Statistica, StatSoft, Inc.).
